# Establishment of discrete element flexible model of the tiller taro plant and clamping and pulling experiment

**DOI:** 10.3389/fpls.2022.1019017

**Published:** 2022-11-03

**Authors:** Liu Wanru, Zhang Guozhong, Zhou Yong, Liu Haopeng, Tang Nanrui, Kang Qixin, Zhao Zhuangzhuang

**Affiliations:** ^1^ College of Engineering, Huazhong Agricultural University, Wuhan, China; ^2^ Key Laboratory of Agricultural Equipment in Mid-Lower Yangtze River, Ministry of Agriculture and Rural Affairs, Wuhan, China

**Keywords:** taro, discrete element flexible model, parameter variation, clamping and pulling force, bench experiment, simulation experiment

## Abstract

The taro harvesting process is affected by a complex system composed of particle mechanics system and multi-body dynamics system. The discrete element method(DEM) can effectively solve the nonlinear problem of the interaction between harvesting components and working materials. Therefore, the discrete element model of taro tiller plants is of great importance for taro harvesting. This paper proposes a simulation method to establish a discrete element flexible plant model and dynamic clamping and pulling process of taro tiller plant. Discrete Element models of taro corm and flexible tiller petiole and leaf were established using DEM method, and the discrete element flexible model of the taro plant was established. Taro clamping and pulling force testing platform was designed and built. The single factor and Plackett-Burman experiments were used to determine the simulation parameters and optimize the taro plant model by taking the correlation coefficient of clamping force and correlation coefficient of pulling force collected from the simulation and the bench experiment as the experiment index. The parameter calibration results of discrete element model of taro plant are as follows: petiole-petiole method/tangential contact stiffness was 8.15×10^9^ N·m^-3^, and normal/tangential critical stress was 6.65×10^6^ Pa. The contact stiffness of pseudostem- corm method was 1.22×10^9^ N·m^-3^, the critical stress of normal/tangential was 1.18×10^5^ Pa, and the energy of soil surface was 4.15×10^6^J·m^-3^. When the pulling speed is 0.1, 0.2, 0.3, 0.4 and 0.5 m·s^-1^, the correlation coefficients between the simulation experiment and the bench experiment are 0.812, 0.850, 0.770, 0.697 and 0.652, respectively. The average value of correlation coefficient is 0.756, indicating that the simulated discrete element plant model is close to the real plant model. The discrete element model of taro plant established in this paper has high reliability. The final purpose of this paper is to provide a model reference for the design and optimization of taro harvester by discrete element method.

## 1 Introduction

Taro is the most important food crop and cash crop of Araceae, and is the staple food of nearly 70 million people in the world (Andreas and Waqainabete, 2018; Aditika et al., 2022). It has a higher starch content than potatoes, sweet potatoes, cassava, etc. (Singla et al., 2020). Taro is native to India (Wang et al., 2012). By 2020, the harvested area of African taro was about 1.6088 million hectares, accounting for 88.91 percent of the world’s planted area. Chinese taro is mainly distributed in the Yangtze River basin, Pearl River Basin and Yellow River basin (Li et al., 2022). Harvesting is an important stage in the process of taro production presently done by digging and clamping. Excavating harvest is a segment-type harvest. First, the thick petiole of the plant is cut off, and then the root and petiole harvester are used to excavate taro corm. This process requires two operations, which is inefficient. Gripper harvesting is combined harvesting, where the belt of the harvester grabs the leaf petiole and pulls the taro out by the roots, then the petiole is cut off by a rotating blade. This way of harvesting can be completed at one time, this way of operation efficiency is high, so the clamping taro harvest is the future harvest taro important way of harvesting (Zhou et al., 2015; Zhu et al., 2022). the research and development cycle of equipment is long, and the cost of processing and trial production is high. Traditional test methods cannot accurately analyze taro plants’ force and movement during harvesting. Therefore. It is of great significance for the optimal design of key components of the taro harvester to study the stress and movement of taro plants under the plant-machine-soil interaction in the process of pulling taro by digital simulation method.

The clamping and pulling mechanical system together with taro plants, soil and other microscopic granular materials constitute a complex physical field system with multi-spatial scale coupling. Evaluating the accuracy of clamping resistance and pulling resistance in taro harvesting has an important impact on the design of the taro harvester, optimization of key components and power system of taro harvesting equipment, and evaluation of operation quality and efficiency. The discrete element method can effectively simulate the nonlinear relationship between the interaction between harvesting components and working materials (Ma et al., 2015; Guo et al., 2021). The key to taro clamping and pulling simulation’s success is using accurate material parameters and particle contact model (Liu et al., 2022). Compared with rice, wheat and maize, corm crops such as taro and potato are also very important food sources for humans. However, the recent research on corm crops using the discrete element method focuses on corm modeling in potato (Liu et al., 2018) and Cyperus edulis root- corm and soil (He et al., 2022). Yang et al. (2022) established a discrete meta-model of cassava petiole and applied it to the simulation of pre-cut cassava planter seeding; Chen et al. (2022) established the discrete element model of cassava seed stem and carried out the simulation test of vibration seed dispersing mechanism. Yu Qingxu et al. established the discrete element model of *Panax notoginseng* seeds and carried out the planting test, which proved that *Panax notoginseng* seeds could be used in the discrete element simulation experiment (Yu, 2019; Yu et al., 2020; Yu et al., 2022). Liu (2021) established the discrete element model of sweet potato corm and carried out the simulation of sweet potato transport device.

Due to the lack of discrete element model of corm crops, the development of contact mechanics model between corm crops and agricultural machinery and various agricultural materials is limited, and the in-depth multidimensional research on the internal mechanism of corm crop-soil-machine and tool interaction has been affected. Therefore, this study’s key is to construct a discrete element flexible model for the taro plant and explore the dynamic mechanical behavior of taro clamping and pulling under plant-machine-soil interaction. Taro plant has anisotropic, inhomogeneous, and petiole tillering characteristics. When using discrete element method to estabish taro plant model, the simple rules of spherical particles cannot be simulated with the complex shape outline rules such as taro particle collision and friction between features, so the discrete element model of the local petioles and corms cannot meet the needs of taro pulling performance simulation. Currently, there is no literature report on the establishing a discrete element model of the taro plant, and its interaction with soil and machine has also not been solved.

This paper proposed a simulation method to establish a discrete element flexible plant model and dynamic clamping and pulling process of taro tiller plant. which can reduce the testing cost and shorten the design process in the structural design of the taro harvester. The remainder of the article is organized as follows. Section 2 introduces the discrete element flexible model of taro tiller plant established by EDEM, and the parameters were calibrated by single factor experiment and Plackett-Burman experiment. The practicability of the taro plant model was verified, and the pulling force of the taro plant were explored in Section 3. Finally, conclusion and the future research directions that can be studied in depth is presented in Section 4.

## 2 Materials and methods

### 2.1 Establishment of discrete element model of taro tiller plants based on EDEM

#### 2.1.1 Establishment of discrete element model of taro corm

**Figure 1:**
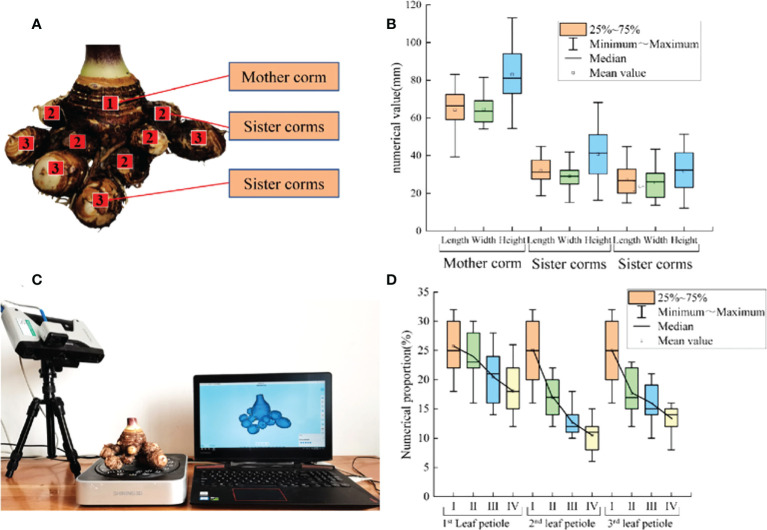
Schematic diagram and size statistics of taro corm. **(A)** The growing position of mother corm, and sister corms; **(B)** The length, width and height of mother corm, and sister corms statistics; **(C)** 3D laser scanning experiment of taro corm; **(D)** The length, width and height of 1st leaf petiole, 2nd leaf petiole and 3rd leaf petiole statistics.

The corms of taro planted by Yanglinggou Taro Cooperative in Hanchuan City, Hubei Province were studied. The geographical location of the area was 113.660221 east longitude and 30.521991 north latitude. Taro corms can be divided into mother corm, sister corms, as shown in **Figure 1A**. Sixty taro samples without damage and disease were collected, and the vernier caliper with an accuracy of 0.01mm was used to measure the physical dimensions of taro corms, and the average values were calculated. The mean length, width, height of the mother corm was 64.26, 64.46 and 83.01mm, respectively. The mean length, width, height of sister corm was 32.06, 29.01 and 40.85mm, respectively, and the mean length, width, height of sister corm was 27.44, 25.62 and 31.74mm, respectively, the statistical results are shown in **Figure 1B**.

3D laser scanning technology can accurately obtain the 3D contour of complex plants. As taro corm is irregular particles with complex shapes. A SHINING 3D Einscan-Pro multi-functional handheld scanner was used to conduct 3D scanning and collect the position coordinates of taro corm in 3D space, 3D laser scanning is shown in **Figure 1C**. Dominik et al. (2021) established an image database of 7 different trees through 3D scanning. Cucinotta et al. (2019) obtained the wear morphology of the plowshare through three-dimensional scanning technology.

**Figure 2:**
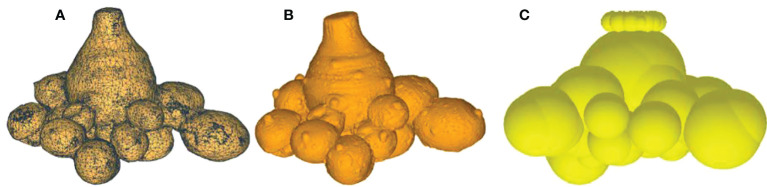
Discrete element model of taro corm. **(A)** Taro corm 3D scanning point cloud model; **(B)** Taro corm contour model; **(C)** Discrete element model of taro corm.

Geomagic Warp software is used for reverse engineering processing to obtain the point cloud data of taro corms, as shown in the **Figure 2A**. The point cloud data is converted into a polygon model to restore the shape contour of taro corms. After trimming the excess surface, deleting the nail, merging, relaxing, smoothing and other operations (Hao et al., 2021), A more accurate polygon model of the taro corm was obtained, as shown in the **Figure 2B**. The discrete element model of the taro corm was established by the fast-filling method of EDEM software, and the number of sphere elements was 58. The discrete element model of taro plant is shown in **Figure 2C**.

The Hertz-Mindlin (no slip) model with high computational accuracy and simulation speed was used among corm particles, and the contact radius between spherical particles:


(1) $$\begin{array}{c} \delta_{i}=r_{1i}+r_{2i}-d_{i} \end{array}$$

Where *δ*_i_ is the contact center distance between two spherical particles, mm; *r*_1i_ is the radius of the first sphere particle, mm; *r*_2i_ is the radius of the second sphere particle, mm; *d*_i_ is the overlap distance in the direction of the connecting line between the center of two spheres, mm.

#### 2.1.2 Establishment of discrete element flexible model for taro petiole

The taro petiole is in the shape of a tiller, and the inner part of the petiole is mainly composed of axial vascular bundle fibers. The diameters of the 1^st^ side petiole, the 2^nd^ side petiole and the 3^rd^ side petiole at different positions from the ground were measured for 60 plants. I stand for 0-5cm from the ground, II for 5-10cm from the ground, III for 10-15cm from the ground, and IV for 15-20cm from the ground, as shown in the **Figure 1D**. The average diameter of the petiole gradually increases from the bottom to the top.

**Figure 3:**
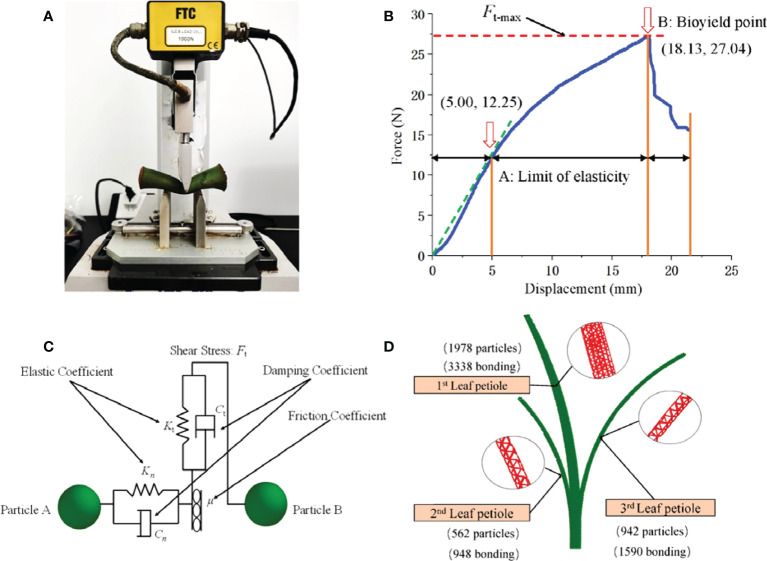
Discrete element model of petiole. **(A)** Three point bending experiment of petiole; **(B)** Displacement-Force of petiole obtained by three-point bending test; **(C)** Discrete element model and particle number of petiole; **(D)** Inter-particle force of taro.

Twenty main petioles were cut at a distance of 5-10 cm from the ground, and the TMS-PRO texture analyzer produced by TFC Company in the United States was used to measure the petiole load-deflection curve at an experiment speed of 60 mm·min-1 (Shen et al., 2015), the instrument accuracy is ±1%, the range is 0~1000 N, and the data acquisition frequency is 50 Hz. The petiole was placed on two horizontal metal supports at the testing machine’s lower end and aligned the sample’s center with the center of the upper clamp; the experiment was started until the petiole was significantly bent, as shown in the **Figure 3A**. where midpoint A is the elastic limit point, and point B is the biological yield point. as shown in the **Figure 3B**. The elastic modulus of the petiole is about 16.69MPa after 20 measurements and the average value is obtained by formula (9). The Poisson’s ratio ψ of the petiole is 0.4 according to the relevant literature (Shi et al., 2018), and the shear modulus of the petiole is 5.96 MPa obtained by formula (10).

The section of the taro petiole can be approximated as a circle, and the moment of inertia *I* of the section relative to the neutral axis is:


(2) $$\begin{array}{c} I=\frac{\pi \left[d_{1}^{4}-{\left(d_{1}-2h\right)}^{4}\right]}{64} \end{array}$$

Where *d*_1_ is the petiole outer diameter, mm; *h* is the petiole wall thickness, mm.

petiole elastic modulus *E*_w_:


(3) $$\begin{array}{c} E_{\text{W}}=\frac{FL_{1}^{3}}{48SI} \end{array}$$

Where *F* is the loading force, N; *L*_1_ is the distance between two supports, mm; *S* is the Bending deflection at the midpoint of the petiole, mm.

Shear modulus *G*_1_ of the petiole:


(4) $$\begin{array}{c} G_{1}=\frac{E}{2(\psi +1)} \end{array}$$

The petiole is subjected to clamping load when the taro plant is pulled from the soil. Combined with the deformation and damage characteristics of the petiole after loading, the Hertz-Mindlin with bonding contact model was selected to reflect the anisotropy and agglomeration characteristics of the petiole, as shown in the **Figure 3C**. the fiber state inside the petiole is characterized by the elastic coefficients *K*_t_, *K*_n_, damping coefficients *C*_t_, *C*_n_ and friction coefficient *μ* between the petiole particles. The model has a total of 5 parameters, the normal bond stiffness *S*_n_ and the tangential bond stiffness *S*_t_ are iterated continuously at unit step intervals and update the load on the bond. The normal critical stress *σ*_max_ and the tangential critical stress *τ*_max_ are the critical threshold for judging whether the cohesive force is broken or not. The cohesive radius is the maximum distance required for the formation of cohesion between particles. A total of 5876 parallel bonding structures were generated between particles, as shown in the **Figure 3D**.

The process model of taro petiole particles from cohesion to failure:


(5) $$\begin{array}{c} \{\begin{array}{c} \;\;\;\;\Delta F_{\delta }=-v_{n}S_{n}A_{s}\Delta t\\ \;\;\;\Delta F_{\alpha }=-v_{t}S_{t}A_{s}\Delta t\\ \;\;\Delta T_{n}=-w_{n}S_{n}J\Delta t\\ \Delta T_{t}=-w_{t}S_{t}J\Delta t \end{array} \end{array}$$

Where *F_δ_* is the normal adhesion force, N; *F_α_* is the tangential adhesion force, N; *T*_n_ is the normal adhesion moment, N·m; *T*_t_ is the tangential adhesion moment, N·m; *v*_n_ is the particle method Vertical velocity, m·s^-1^; *v*_t_ is the particle tangential velocity, m·s^-1^; *w*_n_ is the normal angular velocity, rad·s^-1^; *w*_t_ is the tangential angular velocity, rad·s^-1^. *S*_n_ is the normal bond stiffness, N·mm^-1^; *S*_t_ is the tangential bond stiffness, N·mm^-1^; *A*_s_ is the contact area, m^2^.

The moment of inertia *J* of the parallel bond and the area of the contact area *A*_s_:


(6) $$\begin{array}{c} J=\frac{1}{2}\pi R_{B}^{4} \end{array}$$

(7) $$\begin{array}{c} A_{S}=\pi R_{B}^{2} \end{array}$$

Where *R*_B_ is the bond radius, mm.

Fracture conditions for bond bonds:


(8) $$\begin{array}{c} \{\begin{array}{c} \sigma_{max}<\frac{-F_{\delta }}{A_{S}}+\frac{2T_{t}}{J}R_{B}\\ \tau_{max}<\frac{-F_{\alpha }}{A_{S}}+\frac{2T_{n}}{J}R_{B} \end{array} \end{array}$$

Where *σ*_max_ is the normal critical stress, Pa; *τ*_max_ is the tangential critical stress, Pa.

#### 2.1.3 Establishment of discrete element flexible model for the whole taro plant

The taro leaf does not affect the effect of clamping and pulling. Only its gravity affects the clamping posture. To improve the simulation efficiency, the material parameters of the blade part were equivalent to the petiole, and the Hertz-Mindlin (no slip) contact model was used between the particles.

**Figure 4:**
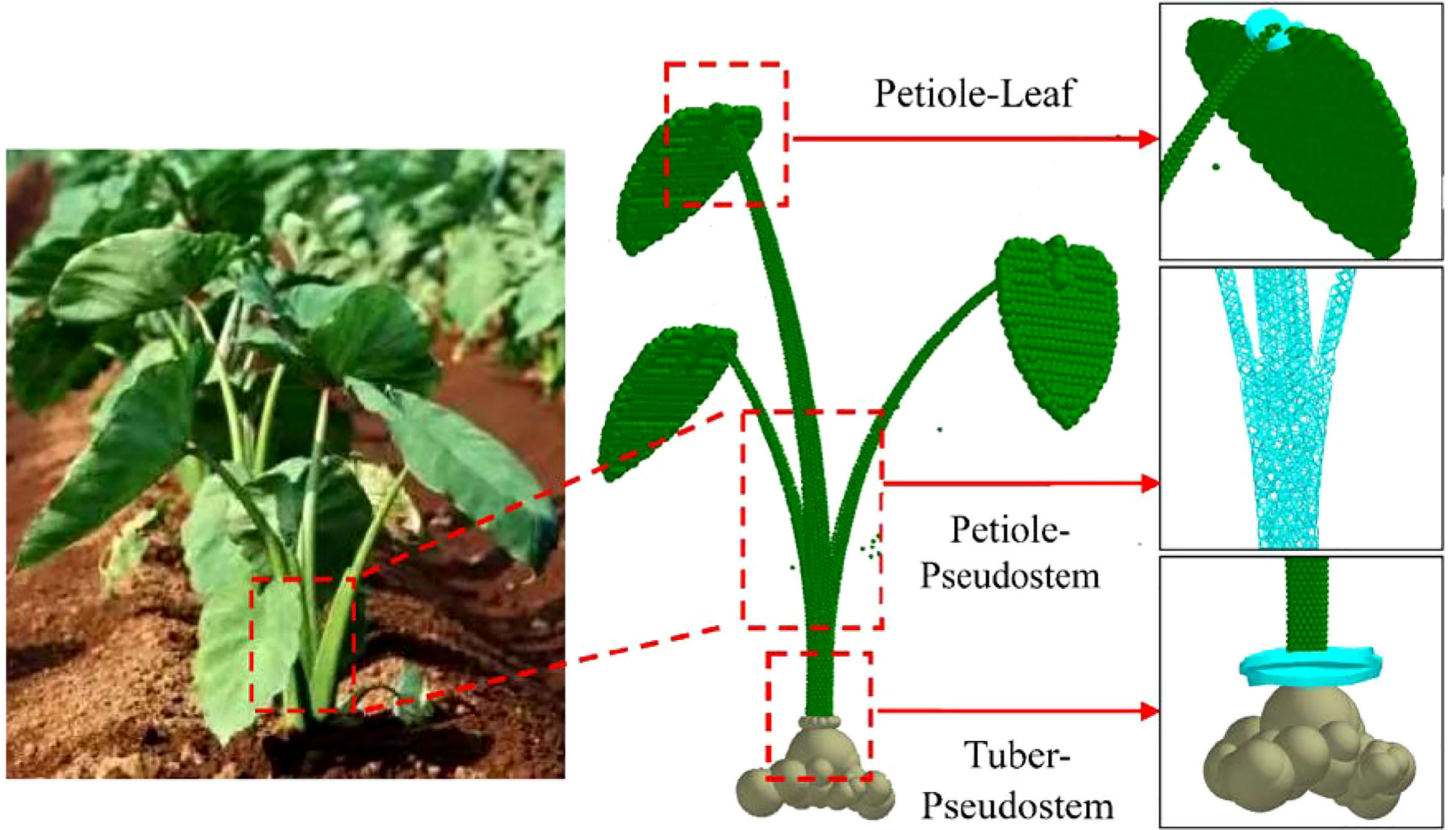
Discrete element flexible model of taro plant.

Taking the corm, petiole and leaf as the aggregation unit, according to the actual shape of the taro plant and the coordinate position of each particle cluster unit, the X, Y and Z axis coordinates of the aggregation unit were located, and the Hertz-Mindlin with bonding particle contact model was used to connect the corm to the petiole and the petiole to the leaf. Micro-unit spherical particles characterize the complex stress-strain characteristics of plants under macroscopic load, then global variables were set, the Fixed Time Step was set to 4.60%, the Cell Size set to 3Rmin, the total number of grids was 3.5×10^6^, and the time was set to 10s. The discrete element model of taro plant established is shown in **Figure 4**.

### 2.2 Taro clamping and pulling force experiment

#### 2.2.1 Construction of taro clamping and pulling force measuring platform

**Figure 5:**
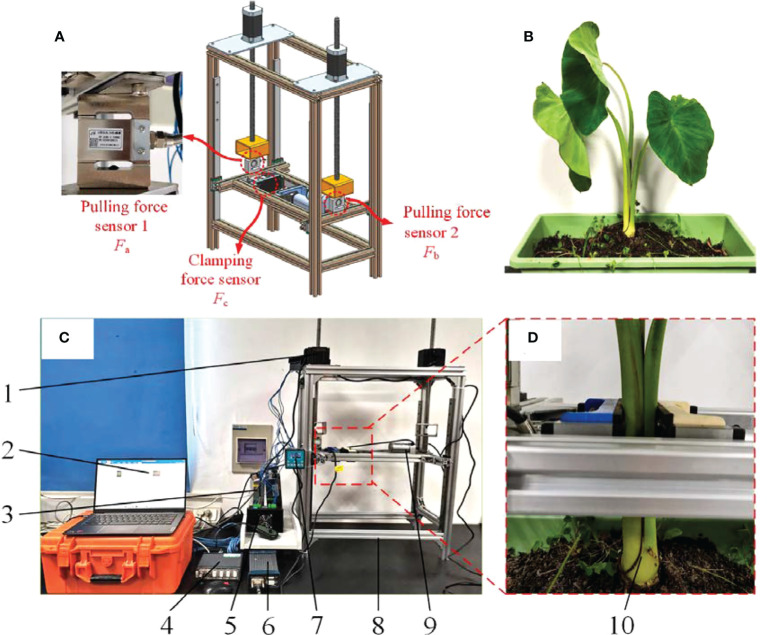
Mechanical force measuring platform of clamping and pulling device. **(A)** Sensor installation location; **(B)** Taro plant; **(C)** Mechanical force measuring platform of clamping and pulling device; 1. 86 stepper motor 2. PC test software 3. 48V power supply 4. DH380 controller 5. Motor driver 6. DH3820 collector 7. 86-stepper motor controller 8. Clamping force measuring platform 9. Electric pusher petiole 10. Taro Plant. **(D)** Clamping position of taro petiole.

In order to explore the actual interaction and dynamic mechanical behavior of taro pulling from the soil, a force measuring device for taro clamping and pulling was designed and fabricated, **Figure 5A** is the 3D model, and **Figure 5C** is the platform. Working principle: Place the taro plant shown in **Figure 5B** in the middle of the left and right clamping boards, Under the action of the electric push rod, the taro petioles are clamped at a constant speed. When the clamping plate is in contact with the taro petiole, the pulling platform was driven by the 86 stepper motor to lift at a constant speed until the petiole does contact with the clamping plate, as shown in **Figure 5D**. Sliding and pulling up the taro plant from the soil, at this time, record the minimum clamping force collected by the clamping platform sensor and the instantaneous pulling resistance collected by the two sensors of the pulling platform.

When the sensor’s sensitivity is 0.0105mv·V^-1^, the dynamic calibration of the sensor is carried out by using weights to eliminate the influence of the vibration of the force measuring platform and the gravity of the bench itself. Finally, complete the process of taro clamping and pulling force measurement

#### 2.2.2 Bench experiment of taro plant clamping and pulling

Use the SDH-1202 fast halogen moisture meter to measure the moisture content of taro corms, petioles and growing soil environment, respectively. Set the drying temperature to 105°C, until the quality no longer changes, and repeat 5 times. The average measured moisture content was 75.42% for corms, 88.34% for petioles, and 21.05% for soil. Take the petiole 5 to 10 cm from the ground as the clamping position, the clamping speed is 12mm·s^-1^, and the pulling speed is 0.3m·s^-1^ as the working conditions, and the petiole does not form with the clamping plate during pulling. The minimum clamping force *F*_C_ is collected at the moment of slippage, and the instantaneous pulling resistance *F*_L1_ and *F*_L2_ collected by the two pull-out sensors are recorded. The sum of *F*_L1_ and *F*_L2_ is recorded as *F*_L_. The mean value of *F*_C_ and *F*_L_ was 300.89N and 201.245N, respectively.

### 2.3 Simulation experiment of taro clamping and pulling

#### 2.3.1 Experimental procedure

When the discrete element method is used to carry out the simulation research of taro clamping and pulling, the accuracy of the model parameters directly affects the data accuracy of the minimum clamping force and the instantaneous pulling resistance. The intrinsic parameters (density, Poisson’s ratio and elastic modulus) are the material’s inherent properties. The necessary parameters for the simulation are obtained through physical experiments and literature results, as shown in **Table 1**. The contact parameters (coefficient of restitution, coefficient of static friction and coefficient of rolling friction) of taro corm and petiole were obtained through tribometer experiment and high-speed photographic experiment in the early stage. The results are shown in **Table 2**. The soil in which taro grows is sandy loam, which has the characteristics of granular material. Hertz-Mindlin with JKR is a contact model based on Hertz theory that can characterize the viscoelasticity between particles (Horabik and Molenda, 2016). Therefore, the Hertz-Mindlin with JKR model was used for the soil, and the Hertz-Mindlin with bonding was used to reflect the adhesion between the petiole and the soil.

**Table 1:** Intrinsic parameters.

Property	Value	Source
Poisson´s ratio of the device	0.3	a, b f
Poisson´s ratio of the corm	0.25	e
Poisson´s ratio of the petiole	0.4	g
Poisson´s ratio of the soil	0.5	c d b
Young’s modulus of the device (Pa)	2.78×1011	a, b f
Young’s modulus of the corm (Pa)	5.30×106	e
Young’s modulus of the petiole (Pa)	1.67×108	Experiment
Young’s modulus of the soil (Pa)	7.50×107	c d b
Density of the device (kg·m-3)	7.80×103	a, b f
Density of the corm (kg·m-3)	1540	e
Density of the petiole (kg·m-3)	1.13×103	Experiment
Density of the soil (kg·m-3)	2.60×103	1 2 d

Parameter source: a: (Liu et al., 2018); b: (Ucgul et al., 2017); c: (Wang et al., 2022); d: (Ucgul and Saunders, 2020); e: (Horabik et al., 2019); f: (Su et al., 2020). g: (Shi et al., 2018).

**Table 2:** Contact parameters.

Property	Coefficient of restitution	Coefficient of static friction	Coefficient of rolling friction
Petiole-Steel	0.1487	0.6135	0.3262
Petiole-Soil	0.1128	0.7054	0.4107
Petiole- Petiole	0.3183	0.5583	0.4267
Pseudostem -Corm	0.0891	0.6073	0.4896
Corm-Steel	0.2769	0.2473	0.3404
Corm -Soil	0.2256	0.4457	0.293
Corm - Corm	0.3257	0.7587	0.6187
Soil-Soil	0.1230	0.3853	0.2670

*S*_n_=*S*_t_, *σ*_max_=*τ*_max_ can simplify the parameter calibration, the inter-particle bonding behavior originates from the liquid bridge between particles (Zhu et al., 2020), and the material moisture content is measured by the inter-particle bonding radius.

Assuming that the material moisture is uniformly distributed, and wrapped around the particle to form a uniform water film, the sum of the thickness of the water film and the particle radius represents the bonding radius. Refer to the existing rhizome agricultural material simulation parameters to set its range, as shown in **Table 3**.

**Table 3:** Parameter range of particle contact model.

Interparticle contact parameters		Ranges
Petiole - Petiole	Normal/tangential contact stiffness X1/(N·m-3)	1.0×109<1.2×1010
	Normal/tangential critical stress X2/(Pa)	5.0×108<1.5×109
Corm - Soil	Normal/tangential contact stiffness X3/(N·m-3)	5.0×105<1.0×107
	Normal/tangential critical stress X4/(Pa)	1.0×104<1.6×105
Soil - Soil	Surface energy X5/(×105J·m-3)	1.0×105<1.0×107

Through the particle filling experiment, the density *ρ* of the filling particles is obtained by correcting the bulk density of the material, and the formula is:


(9) $$\begin{array}{c} \rho =\frac{3V}{4\pi kR_{i}^{3}}\rho_{i} \end{array}$$

Where *ρ* is the density of filling particles, kg·m^-3^; *V* is the volume of the container, m^3^; *K* is the number of filling particles, each; *R*_i_ is the radius of the filling particles, mm; *ρ*_i_ is the bulk density of the material, kg·m^-3^.

Volume *V*_s_ of filled spherical particles:


(10) $$\begin{array}{c} V_{S}=\frac{4}{3}\pi r^{3} \end{array}$$

Combining (9) and (10) yields:


(11) $$\begin{array}{c} \rho =\frac{V}{kV_{S}}\rho_{i} \end{array}$$

The total volume *V*_i_ occupied by the material particles is:


(12) $$\begin{array}{c} V_{i}=KV_{\text{s}} \end{array}$$

Therefore, the bulk density *ρ*_i_ of the material:


(13) $$\begin{array}{c} \rho_{i}=\frac{\rho V_{i}}{V} \end{array}$$

Then the total weight *m*_i_ of material particles is:


(14) $$\begin{array}{c} m_{i}=\rho_{i}V_{i}=\frac{4}{3}\pi R_{i}^{3}\rho_{i} \end{array}$$

The total volume of water in the material *V*_w_:


(15) $$\begin{array}{c} V_{w}=\frac{4}{3}\pi R_{B}^{3}-\frac{4}{3}\pi R_{i}^{3} \end{array}$$

According to the material moisture content *w*:


(16) $$\begin{array}{c} w=\frac{\left(\frac{4}{3}\pi R_{B}^{3}-\frac{4}{3}\pi R_{i}^{3}\right)\rho_{2}}{\frac{4}{3}\pi R_{i}^{3}\rho_{1}} \end{array}$$

Derivation of formula (13) yields the bonding radius *R*_B_:


(17) $$\begin{array}{c} R_{B}=\sqrt[{3}]{\frac{\rho_{2}+w\rho_{1}}{\rho_{2}}} \end{array}$$

Where, *w* is the moisture content of the material, %; *R*_B_ is the bonding radius between particles, mm; *ρ*_i_ is the bulk density of the material, kg·m^-3^; *ρ*_2_ is the density of water, kg·m^-3^; *R*_i_ is the filling material particles Radius, mm; *V*_s_ is the filling ball particle volume, m^3^; *V*_i_ is the total volume occupied by material particles, m^3^; *m*_i_ is the total weight of material particles, kg; *m*_w_ is the total moisture weight, kg; *V*_w_ is the total volume of water in material, m^3^.

**Figure 6:**
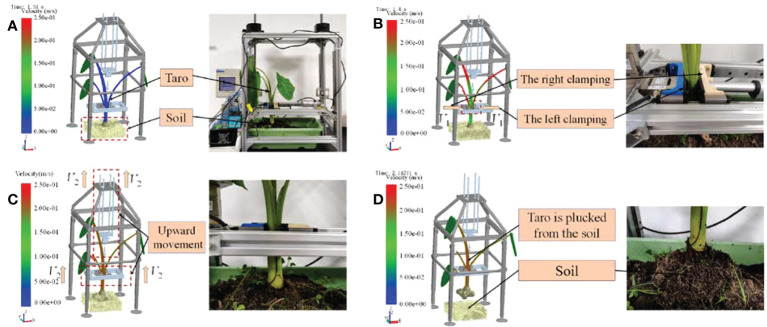
DEM simulation process. **(A)** The taro petiole is placed in the middle of the gripper; **(B)** The left and right clamping boards clamp the taro petiole; **(C)** The ascending mechanism lifts the taro plant; **(D)** Taro is completely pulled from the soil.

The virtual prototype model of the pulling device established by NX.12.0 is saved as stl format and imported into EDEM. In order to facilitate simulation and calculation, the parts that are not related to contact are removed, and the discrete element model of the taro clamping and pulling device is established. The size of the clamping plate is 100×50mm. The target save interval is set to 0.005s, the total simulation time is 3s, the gravitational acceleration is in the negative direction of the Z axis, and the value is -9.8kg·m^-3^. The main operation process Includes the following 4 steps:

The actual growth depth of taro in the soil is about 15cm underground, and the surface is exposed to about 1cm of soil. Therefore, according to the actual growth situation, the taro plants are grown in a soil trough with a size of 300 × 300 × 200 mm, and a total of 37875 particles are generated in the soil trough. The position of the clamping experiment platform was adjusted, and the two clamping plates were fixed at the petiole 5<10 cm from the ground, as shown in **Figure 6A**.

The left and right clamping plates speed were set to 12 mm·s^-1^ in the X-axis to clamp the petiole until the clamping plates on both sides are in contact with the petiole, as shown in **Figure 6B**.

The clamping plates continue to clamp the petiole, and the left and right clamping plates and the pulling platform are set at a speed of 0.3m·s^-1^ in the Z-axis direction to pull up the plants until the clamping plates do not slip with the petioles. Stop the speed of the clamping plate in the X-axis direction at this time, record the force change of the clamping plate in the X-axis direction, and the clamping force collected at the moment of slip is recorded as the clamping force *F*_C_ for pulling out the taro, as shown in **Figure 6C**.

Continue to pull up the plants in the Z-axis direction until the plants are completely pulled up from the soil and record the pulling platform at the force change in the Z-axis direction, which is recorded as *F*_L_. as shown in **Figure 6D**.

#### 2.3.2 Experiment index

Bench experiment to measure the holding force of the *F*_a_, pulling force for *F*_b_, simulation experiment of clamping force for the *F*_C_, pulling force for *F*_L_, respectively using Origin data processing software simulation experiment bench experiment and measurement of two curves for data analysis, the correlation coefficient of the two clamping force curve for *R*_C_, the correlation coefficient of the pulling force of two curve for *R*_L_, The larger the correlation coefficient is, the higher the similarity of the two curves is, indicating that the tension measured in the simulation experiment is closer to the tension obtained in the actual experiment. *R*_C_ and *R*_L_ are calculated according to equations (18) and (19) respectively:


(18) $$\begin{array}{c} R_{C}=\frac{Cov(F_{a},F_{C})}{\sigma_{F_{a}}\sigma_{F_{C}}} \end{array}$$

(19) $$\begin{array}{c} R_{L}=\frac{Cov(F_{b},F_{L})}{\sigma_{F_{b}}\sigma_{F_{L}}} \end{array}$$

Where, *F*_a_ =300.89N; *F*_b_ =201.245N.

#### 2.3.3 Single factor simulation experiment

In order to ensure the reliability of the discrete element flexible model of the whole taro plant, the Hertz-Mindlin with bonding model parameters between petiole, corm and soil, and Hertz-Mindlin with JKR model parameters between soils were calibrated. Taking the normal/tangential contact stiffness *X*_1_ between petiole, the normal/tangential critical stress *X*_2_, the normal/tangential contact stiffness *X*_3_ between corm and soil, the normal/tangential critical stress *X*_4_, and the inter-soil surface energy *X*_5_ as for the experimental factors, single-factor simulation experiments were carried out with *R*_C_ and *R*_L_ as the experimental indicators. The experimental parameter levels are shown in **Table 4**.

**Table 4:** Value range of single factor exposure parameters.

Contact parameters		Level	Fixed value
Petiole- Petiole	X1/(N·m-3)	1.0×109, 4.0×109, 6.0×109, 8.0×109, 1.0×1010, 1.2×1010	X2 = 1.1×109, X3 = 6.5×106, X4 = 1.0×105, X5 = 6.0×106
	X2/(Pa)	5.0×108, 7.0×108, 9.0×108, 1.1×109, 1.3×109, 1.5×109	X1 = 0.8×109, X3 = 6.5×106, X4 = 1.0×105, X5 = 6.0×106
Corm - Soil	X3/(N·m-3)	5.0×105, 2.5×106, 4.5×106, 6.5×106, 8.5×106 1.0×107	X1 = 0.8×109, X2 = 1.1×109, X4 = 1.0×105, X5 = 6.0×106
	X4/(Pa)	1.0×104, 4.0×104, 7.0×104, 1.0×105, 1.3×105, 1.6×105	X1 = 0.8×109, X2 = 1.1×109, X3 = 6.5×106, X5 = 6.0×106
Soil- Soil	X5/(×105J·m-3)	1.0×105, 2.0×106, 4.0×106, 6.0×106, 8.0×106, 1.0×107	X1 = 0.8×109, X2 = 1.1×109, X3 = 6.5×106, X4 = 1.0×105,

#### 2.3.4 Plackett-Burman experiment

The Plackett-Burman calibration method is accurate and efficient and has been widely used in discrete element parameter calibration (Xia et al., 2019; Fang et al., 2022). First, based on the single factor experiment results, each factor is increased in equal steps to create each step parameter level and carry out a simulation experiment. Then, the parameters are further reduced through the changing trend of the Correlation coefficient of clamping force *R*_C_ and the Correlation coefficient of pulling force *R*_L_, Finally, a multi-factor regression model was established, the maximum optimal solution of *R*_C_ and *R*_L_ was calculated, and parameter calibration was completed. Single factor simulation experiment parameters with correlation coefficient more than 50% were used as the range of Plackett-Burman experiment parameters, *X*_1_ (6.0×10^9^<1.0×10^10^ N·m^-3^), *X*_2_ (9×10^8^<1.5×10^9^ Pa), *X*_3_ (4.5×10^6^<8.5×10^6^ N·m^-3^), *X*_4_ (7.0×10^4^<1.6×10^5^ N·m^-3^), *X*_5_ (2.0×10^6^<6.0×10^6^ N·m^-3^).

## 3 Results and discussion

### 3.1 Parameter calibration of discrete element model of taro whole plant

#### 3.1.1 Analysis of single factor experiment results

**Figure 7:**
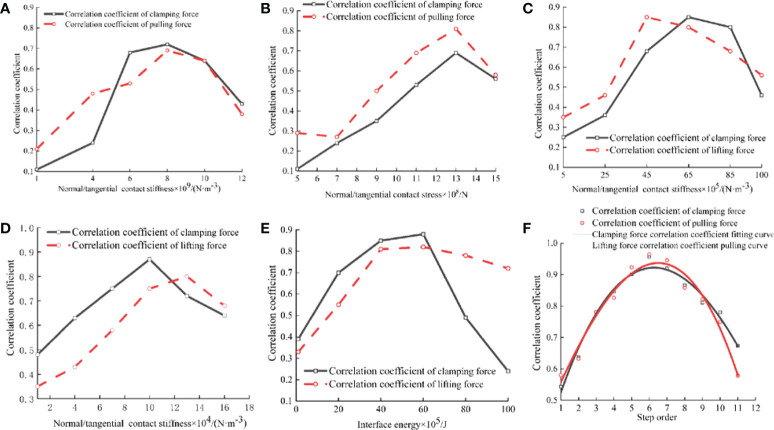
Results of Single factor and Plackett-Burman simulation experiments. **(A)** Changes of RC and RL at different X1 levels; **(B)** Changes of RC and RL at different X2 levels; **(C)** Changes of RC and RL at different X3 levels; **(D)** Changes of RC and RL at different X4 levels; **(E)** Changes of RC and RL at different X5 levels; **(F)** Fitting plot of step order and correlation coefficient.

With the increase of *X*_1_ and *X*_2_, both *R*_C_ and *R*_L_ show a trend of first increasing and then decreasing, as shown in **Figures 7A, B**, mainly because the cross-section of the petiole is composed of many fiber tubes, and there are a large number of fiber tubes around them. For parenchyma cells, the fibrous tube bundles shrink when they are clamped, and the culm is deformed. When pulling up, friction occurs between the clamping plates on both sides and the epidermis of the culm. Therefore, *X*_1_ and *X*_2_ will affect both *R*_C_ and *R*_L_. With the increase of *X*_3_ and *X*_4_, both *R*_C_ and *R*_L_ showed a trend of first increasing and then decreasing, as shown in **Figures 7C, D**, With the increase of *X*_5_, both *R*_C_ and *R*_L_ showed a trend of first increasing and then decreasing, as shown in **Figure 7E**. The Plackett-Burman experiment was carried out in the range of experimental factor data with correlation coefficient more than 50% in the single factor experiment.

#### 3.1.2 Analysis of Plackett-Burman experiment results

The starting point parameter group of Plackett-Burman experiment climbing was *A*_1_ (the lower limit of the contact parameter to be calibrated), and the end parameter group was *A*_m_ (the higher limit of the contact parameter to be calibrated), then each step parameter group can be expressed as:


(20) $$\left[\begin{array}{l} A_{1}\\ A_{2}\\ A_{3}\\ \;\cdot \\ \;\cdot \\ \;\cdot \\ A_{m} \end{array}\right]=\left[\begin{array}{l} X_{11}\;X_{12}\cdots X_{1n}\\ X_{21}\;X_{22}\cdots X_{2n}\\ X_{31}\;X_{32}\cdots X_{3n}\\ \;\cdot \\ \;\cdot \\ \;\cdot \\ X_{m1}\;X_{m2}\cdots X_{mn} \end{array}\right]=\left[\begin{array}{l} \;A_{1}\\ A_{1}+A\\ A_{1}+2A\\ \;\cdot \\ \;\cdot \\ \;\cdot \\ A_{1}+(m-1)A \end{array}\right]$$

Where, *m* is the total number of steps for climbing (that is, the number of calibration experiments), *n* is the total number of parameters to be calibrated (*n*=5), and the step size *A* is:


(21) $$\begin{array}{c} A=\frac{A_{m}-A_{1}}{m-1} \end{array}$$

The value range of step order *x* is 1≤*x*≤*m*, and the value corresponding to each contact parameter group:


(22) $$\begin{array}{c} A_{x}=(x-1)A+A_{1} \end{array}$$

(23) $$\begin{array}{c} E_{C}=f_{C}(X_{1},X_{2},X_{3},X_{4},X_{5}) \end{array}$$

(24) $$\begin{array}{c} E_{L}=f_{L}(X_{1},X_{2},X_{3},X_{4},X_{5}) \end{array}$$

The larger *m* is, the closer the contact parameters of each group of calibration experiments are, and the clamping force *F*_C_ and the pulling resistance *F*_L_ will be more accurate with the change of the parameter group, but the number of simulations will increase. Based on the calibration accuracy and workload, this research uses Design Expert software to design 11 groups of experiments including 5 influencing factors and divides the high and low levels of each influencing factor into 10 equally for the steepest climbing experiment. The experimental design is shown in **Table 5**.

**Table 5:** Calibration experiment results.

Step order/x	X1/×109	X2/×108	X3/×106	X4/×104	X5/×106	FC/N	RC	FL/N	RL
1	6.00	9.00	4.50	7.00	2.00	554.23	0.54	347.03	0.58
2	6.40	9.60	4.90	7.90	2.40	472.35	0.64	318.38	0.63
3	6.80	10.20	5.30	8.80	2.80	385.81	0.78	257.68	0.78
4	7.20	10.80	5.70	9.70	3.20	355.91	0.85	243.70	0.83
5	7.60	11.40	6.10	10.60	3.60	333.84	0.90	217.99	0.92
6	8.00	12.00	6.50	11.50	4.00	314.48	0.96	208.67	0.96
7	8.40	12.60	6.90	12.40	4.40	326.98	0.92	212.82	0.95
8	8.80	13.20	7.30	13.30	4.80	347.49	0.87	234.58	0.86
9	9.20	13.80	7.70	14.20	5.20	371.24	0.81	245.09	0.82
10	9.60	14.40	8.10	15.10	5.60	385.76	0.78	268.68	0.75
11	10.00	15.00	8.50	16.00	6.00	447.02	0.67	347.87	0.58

With the increase of step order, both *R*_C_ and *R*_L_ show a trend of first increasing and then decreasing. The step order and correlation coefficient are fitted, as shown in **Figure 7F**. The fitting equations:


(25) $$\begin{array}{c} R_{C}=3.18\times 10^{-4}x^{3}-0.02x^{2}+0.20x+0.35 \end{array}$$

(26) $$\begin{array}{c} R_{L}=-5.2\times 10^{-4}x^{3}-0.005x^{2}+0.14x+0.43 \end{array}$$

Among them, the coefficient of determination $R_{c}^{2}=96.65\%$ , $R_{L}^{2}=95.06\%$ , the fitting degree of the fitting equation to the simulation experiment value is good.

According to the minimum value of the quadratic fitting parabola, when the step order *x*_1 =_ 6.48, *x*_2 =_ 6.27, the *R*_C_ and *R*_L_ values are the maximum. *x*_1_ and *x*_2_ mean *x*=6.375, at this time *R*_C_=0.865, *R*_L_ =0.937. According to formula (22), the final result of the contact parameter group corresponding to the interaction of the taro plant: *X*_1_ (8.15×10^9^ N·m^-3^), *X*_2_ (1.22×10^9^ Pa), *X*_3_ (6.65×10^6^ N·m^-3^), *X*_4_ (1.18×10^5^ N·m^-3^), *X*_5_ (4.15×10^6^ N·m^-3^).

### 3.2 Validation of the taro tiller plant model

To verify the application of the taro tiller plant model, Substitute *X*_1_, *X*_2_, *X*_3_, *X*_4_, *X*_5_ into the simulation experiment, The clamping speed was set to 12mm·s^-1^, and the pulling speed was set to 0.3 m·s^-1^, the clamping plate was set at 5<10 cm from the soil surface at the petiole, and the minimum clamping force *F*_C_ and the instantaneous pulling resistance *F*_L_ at the critical moment of slippage during the pulling process of the taro are recorded.

**Figure 8:**
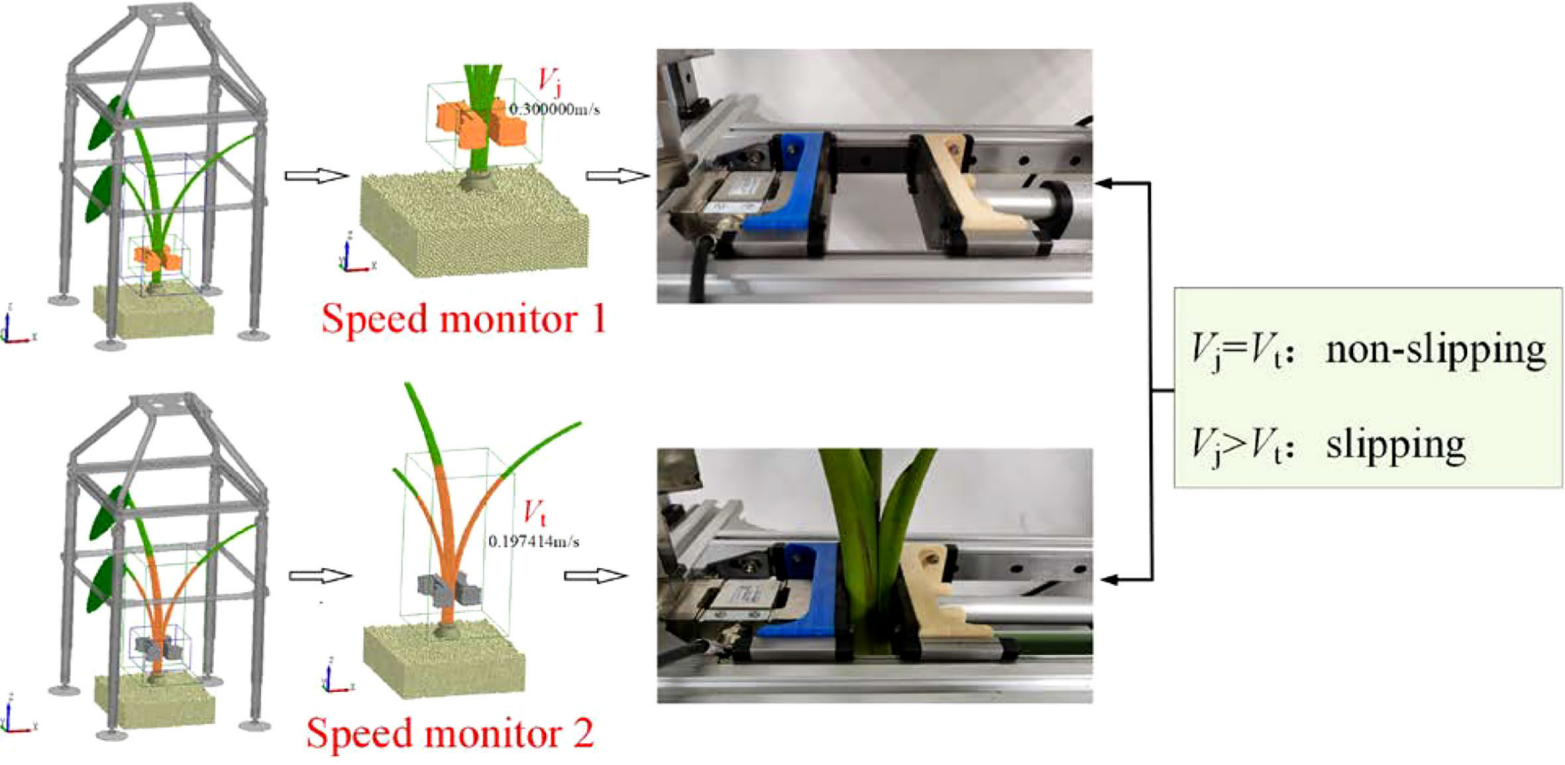
The speed monitor established by the simulation experiment.

The relative velocity of the petiole and the clamping plate in the pulling direction is an important factor to judge whether the two have slipped. Therefore, combined with the post-processing function of EDEM, two local relative velocity monitoring sensors are established at the petiole clamping position. To monitor the movement speed of the holding plate and the taro plant in the pulling direction, as shown in **Figure 8**, output the speed changes of the petiole and the holding plate in the two monitors at each time step, calculated by formula (27) Obtain the relative motion speed between the petiole and the clamping plate:


(27) $$\begin{array}{c} \eta_{i}=\frac{V_{j}}{V_{e}} \end{array}$$

where *η*_i_ is the relative velocity of the petiole and the clamping plate in the pulling direction at the *i* moment, m·s^-1^; *V*_j_ is the speed of the clamping plate in the pulling direction, m·s^-1^; *V*_e_ is the taro plant in the pulling direction. The speed in the pulling direction, m·s^-1^.

When *η*_i_>1, it means that the petiole and the clamping plate have slipped, and the clamping force will continue to be applied at this time; until *η*_i_=1, it means that the petiole and the clamping plate are not affected by the sliding force and record this time. It is the *F*_C_ for taro to pull out, and under this clamping force, the *F*_L_ of taro is measured synchronously.

### 3.3 Comparison of bench experiment and simulation experiment

**Figure 9:**
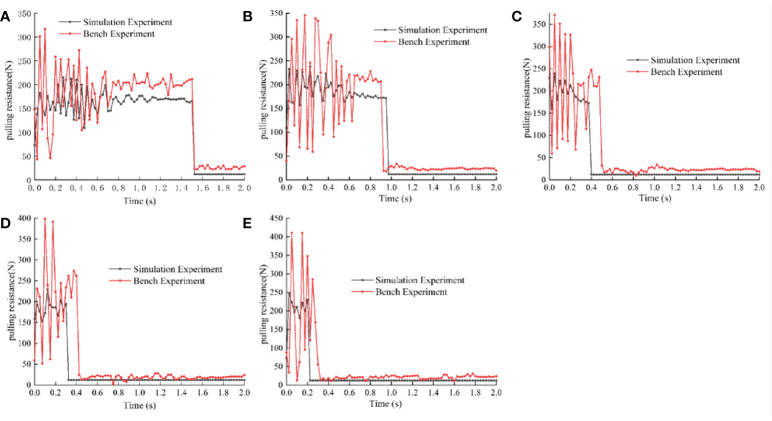
Comparison of bench experiment and simulation experiment. **(A)** Comparison between the bench and simulation when the pulling speed is 0.1m·s-1; **(B)** Comparison between the bench and simulation when the pulling speed is 0.2m·s-1; **(C)** Comparison between the bench and simulation when the pulling speed is 0.3m·s-1; **(D)** Comparison between the bench and simulation when the pulling speed is 0.4m·s-1; **(E)** Comparison between the bench and simulation when the pulling speed is 0.5m·s-1.

When the clamping force of the simulation experiment and bench experiment set into a fixed value 300 N, respectively, in the speed of 0.1, 0.2, 0.3, 0.4, 0.5 m·s^-1^ under the condition of experiment, we got the data from 10 groups of pulling, the same speed of simulation experiment data and bench data on the same picture, as shown in **Figure 9**. We know from the experiment data that the pulling variation trend of the simulation experiment and the bench experiment is basically the same, but the pulling value of the bench experiment is larger than that of the simulation experiment, because the environment of the simulation experiment is more ideal than that of the bench experiment. By comparing the result of the same experiment method under the condition of different speed, it is found that with increasing speed, pulling fluctuation frequency is reduced, but the volatility increases, this is because the impact of the speed, the greater the taro plants are bigger, and it also can be faster from the soil, but the petioles is at risk of being destroyed. We successively conducted correlation analysis on different experiments at the same speed and obtained that the correlation coefficients of pulling variation between simulation experiment and bench experiment were 0.812, 0.850, 0.770, 0.697 and 0.652, respectively. The average value of correlation coefficients was 0.756, which indicated that the discrete element plant model established by simulation was close to the real plant. The discrete element model of taro plant established in this paper has high reliability.

## 4 Conclusion

The discrete element flexible model of taro plant was established by using EDEM software, and a method to test the clamping and pulling resistance of taro in the harvesting process was proposed, which provided a theoretical basis and model reference for the research and development of taro harvesting machinery. The parameter calibration results of discrete element model of taro plant are as follows: petiole-petiole method/tangential contact stiffness was 8.15×10^9^ N·m^-3^, and normal/tangential critical stress was 6.65×10^6^ Pa. The contact stiffness of pseudostem- corm method was 1.22×10^9^ N·m^-3^, the critical stress of normal/tangential was 1.18×10^5^ Pa, and the energy of soil surface was 4.15×10^6^J·m^-3^.

When the pulling speed is 0.1, 0.2, 0.3, 0.4 and 0.5 m·s^-1^, the correlation coefficients between the simulation experiment and the bench experiment are 0.812, 0.850, 0.770, 0.697 and 0.652, respectively. The average value of correlation coefficient is 0.756, indicating that the simulated discrete element plant model is close to the real plant model. The discrete element model of taro plant established in this paper has high reliability.

In the future, the discrete element flexible model of taro plants can be applied to many aspects - studying the reaction force of taro corms and soil slip process when the tractor walking tire compacts the soil; carrying out the simulation experiment of the cutting process of petiole, which is used for the structural design of the cutting blade and the determination of important parameters such as the cutting angle. It can also provide a basis for the structural design of the excavating shovel based on discrete element and multi-body dynamics methods, and at the same time provide an important theoretical model for the in-depth study of corm excavation damage.

## Data availability statement

The original contributions presented in the study are included in the article/supplementary material. Further inquiries can be directed to the corresponding author.

## Author contributions

LW, ZG and ZY designed and performed the experiments and analyzed the data. LW, LH and TN wrote the manuscript. KQ and ZZ made the pictures. All authors contributed to the article and approved the submitted version.

## Funding

This study was supported by the National Characteristic Vegetable Industry Technology System Special Project, Project, No. CARS-24-D-02.

## Acknowledgments

We thank the Engineering Training Center of Huazhong Agricultural University for providing the test site.

## Conflict of interest

The authors declare that the research was conducted in the absence of any commercial or financial relationships that could be construed as a potential conflict of interest.

## Publisher’s note

All claims expressed in this article are solely those of the authors and do not necessarily represent those of their affiliated organizations, or those of the publisher, the editors and the reviewers. Any product that may be evaluated in this article, or claim that may be made by its manufacturer, is not guaranteed or endorsed by the publisher.
